# CATIE and CUtLASS (UK): Is it time psychiatrists start changing their practice?- The debate goes on!

**DOI:** 10.4103/0019-5545.49464

**Published:** 2009

**Authors:** Rupak Dasgupta, Charu Dasgupta

**Affiliations:** Working in Forensic Learning Disabilities at Calderstones NHS Trust, Lancashire, UK. E-mail: rupak2charu@yahoo.co.in

Sir,

In the year 2005 a powerful double blinded trial named CATIE (U.S. Clinical Antipsychotics Trials of Intervention Effectiveness) was published in New England Journal of Medicine by *Lieberman et al*.[1] This was an 18 month trial which showed most patients in each treatment group that is olanzapine or quetiapine or risperidone or ziprasidone or perphenazine,discontinued the assigned treatment because of lack of effect or intolerability. In 2006, *Jones et al,*[2] published the famous UK CUtLASS study which showed that the second generation antipsychotics had no clear advantage over first generation antipsychotics and thus shook the basic pillar of NICE guidelines for treatment of first onset schizophrenia.Both the trials were multicentred, pragmatic,double blinded RCTs and hence their findings need serious consideration.

In the CUtLASS study the QoL was measured only for a period of 1 year which is too less a time to assess QoL longitudinally. In CUtLASS, the first and second generation antipsychotic arms showed less than expected participants which was 64 and71 in each respectively,with a power of 75%. It was originally estimated at the beginning of the study that at least 110 participants would be required in each arm to get a power of 80%.

In the CUtLASS study many psychiatrists chose sulpiride in the first generation arm which has a very similar (though this is controversial) side effect profile to second generation antpsychotics. Also clinically we know that we are seeing far less cases of tardive dyskinesia than before. It is not clear however whether it is because of the use of second generation antipsychotics now or because high dose of typicals that were used in the past. But at the same time these studies point to the hypothesis that psychiatrists as community have been influenced by a systematic campaign from pharmaceutical companies which has greatly increased the cost of treatment in schizophrenia, when the same money could have been spent in stronger areas of need in this client group like - therapy, rehabilitation, support in day centres, housing, life skills training etc. In conclusion the strength of this hypothesis has to be tested in times to come and further studies are needed which observe patients more longitudinally than cross-sectionally.
